# Mr. Teale's *Case of Secondary Small-Pox*

**Published:** 1812-12

**Authors:** Thomas Teale

**Affiliations:** Member of the Royal College of Surgeons. Leeds


*To the Editors of the Medical and Physical Journal.*

GENTLEMEN,

A CASE of secondary Small-pox having lately occurred in a young gentleman, my apprentice, I take the liberty of sending it for insertion in your Journal, if you think it of sufficient practical utility.

Mr. William Marsden, aged 15, son of the late Rev. Thomas Marsden, of Kildwick in Craven, became affected with slight symptoms of fever about the 19th of August last, which gradually increased till the 22d. Towards evening of that day, an eruption appeared, in distinct spots, which I imagined would prove to be the chicken-pox; finding, upon inquiry, that he was inoculated when young for the small-pox, and had the disease severely.

Upon examining his arms, I found two large cicatrices upon one arm, and one upon the other.

The symptoms of fever continued; the pustules maturated kindly, went through the regular stages of small-pox, and did not begin to dry upon the face until the seventh day. On the eighth day of the eruption, I inoculated, from one of the pustules, an infant, which sickened at the usual period, and had the distinct small-pox in a very satisfactory manner. Dr. Carr saw the patient daily, and the case was inspected by Dr. Thorp, Messrs. Key, (senior and junior,) Logan, Cass, and others, all of whom pronounced it to be a case of genuine small-pox.

The disease which he had in his infancy from inoculation, seems to have been equally decisive in its character. I found, upon inquiry, that at two years old he was inoculated by Dr. (then Mr.) Moorhouse, of Skipton in Craven, whose present advanced age prevents my obtaining the necessary information from him; but Mr. Hardacre, surgeon, of Gargrave, who was, at that time, Dr. Moorhouse's assistant, informs me, by letter, that "there cannot remain the least shadow of doubt but that he had the small-pox in a regular manner," although at this distance of time he is unable to give me the exact particulars. He refers me, however, to Miss Midgley, a lady who was niece to Mr. Marsden, and who made a part of the family at that time. Miss M. has had the goodness to give me a very clear account of the disease, and says that she sat with him upon her knee for two days and nights; that he had a very considerable eruption, and was so ill as to be considered in great danger.

Mrs.

Mrs. Marsden (his mother) has a perfect recollection of the circumstances, and in every particular confirms his account. I have the honor to be, Gentlemen,

Your most obedient humble Servant,

*Leeds,* THOMAS TEALE,

*Nov.* 9, 1812. Member of the Royal College of Surgeons.

